# Channel component correlation analysis for multi-channel EEG feature component extraction

**DOI:** 10.3389/fnins.2025.1522964

**Published:** 2025-04-02

**Authors:** Wenqiang Yan, Qi Luo, Chenghang Du

**Affiliations:** ^1^School of Mechanical Engineering, Xi’an Jiaotong University, Xi’an, China; ^2^State Key Laboratory of Robotics, Shenyang Institute of Automation, Chinese Academy of Sciences, Shenyang, China

**Keywords:** channel component correlation analysis, electroencephalogram (EEG), feature component extraction, multi-channel signal, event-related potentials (ERP)

## Abstract

**Introduction:**

Electroencephalogram (EEG) analysis has shown significant research value for brain disease diagnosis, neuromodulation and brain-computer interface (BCI) application. The analysis and processing of EEG signals is complex since EEG are nonstationary, nonlinear, and often contaminated by intense background noise. Principal component analysis (PCA) and independent component analysis (ICA), as the commonly used methods for multi-dimensional signal feature component extraction, still have some limitations in terms of performance and calculation.

**Methods:**

In this study, channel component correlation analysis (CCCA) method was proposed to extract feature components of multi-channel EEG. Firstly, empirical wavelet transform (EWT) decomposed each channel signal into different frequency bands, and reconstructed them into a multi-dimensional signal. Then the objective optimization function was constructed by maximizing the covariance between multi-dimensional signals. Finally the feature components of multi-channel EEG were extracted using the calculated weight coefficient.

**Results:**

The results showed that the CCCA method could find the most relevant frequency band between multi-channel EEG. Compared with PCA and ICA methods, CCCA could extract the common components of multi-channel EEG more effectively, which is of great significance for the accurate analysis of EEG.

**Discussion:**

The CCCA method proposed in this study has shown excellent performance in the feature component extraction of multi-channel EEG and could be considered for practical engineering applications.

## 1 Introduction

Electroencephalogram (EEG) reflects the electrophysiological activity on the cerebral cortex or scalp’s surface, which includes valuable information related to physiological state and disease. In clinical studies, EEG signals can be used to diagnose certain brain diseases and provide effective treatments for some of these diseases (Dockree et al., 2017; Tokariev et al., 2019; Wessel et al., 2016; Martínez et al., 2019; Hamilton et al., 2019). In engineering applications, EEG-based brain-computer interfaces (BCIs) have been developed. Because different mental activities lead to typical, distinguishable, and task-specific patterns of EEG signals, a BCI system can be used to achieve control goals by extraction and classification of EEG features (Anumanchipalli et al., 2019; Heelan et al., 2019; Kubanek et al., 2020; Yan et al., 2018; Edelman et al., 2019). However, EEG signals are nonstationary random signals without ergodic states and often contain strong background noise. Therefore, the analysis and processing of EEG signals are complex.

EEG are collected by electrodes arranged in different brain regions, possessing multi-channel characteristic. Due to the inevitable existence of information redundancy in EEG, extracting feature components effectively from multi-channel EEG has become a critical step in EEG analysis. Principal component analysis (PCA) (Rahman et al., 2020; Rahman et al., 2019) and independent component analysis (ICA) (Feng et al., 2020; Zhua et al., 2020) are the most commonly used multi-channel EEG feature component extraction methods. PCA reduces the dimensionality of the data space to eliminate information redundancy and noise by keeping only those characteristics that contribute most to its variance. A set of orthogonal basis vectors is determined by PCA as the linear combination with maximum variance. The weights and eigenvectors can be calculated through an orthogonal linear transformation of data where the eigenvectors constitute the new axes, resulting in an orthogonal and optimal coordinate system. PCA assumes that the useful signal and noise are statistically uncorrelated. However, this lack of correlation with noise is not necessarily true for multi-channel EEG signals, so PCA often behaves poorly in feature component extraction for these signals. In ICA, a multi-channel observed signal is decomposed into a set of linearly independent signals. ICA assumes statistical independence of the signal sources and the non-Gaussian nature of independent components, and aims to separate independent signals from mixed signals. The objective criterion function of ICA is defined as the measurement of the independence of the independent components (ICs). Many criteria have been proposed, such as minimization of mutual information (MMI), infomax (or maximization of entropy, ME), maximization of negentropy (MN), and maximum likelihood estimation (MLE) (Bell and Sejnowski, 1995; Lee et al., 1999; Cardoso, 1997; Yang and Amari, 1997). ICA performs quite well in blind source separation (BSS) of electrophysiological signals due to the uniqueness of independent components. However, ICA requires an additional process to determine which of the independent components are useful signals, and there is still no widely accepted and effective method to distinguish noise and useful signals from the ICs.

The essence of extracting effective feature components from multi-channel EEG is to extract common components in multi-channel signals, which are often in a specific frequency band. Therefore, this study firstly decomposed each channel signal in multi-channel EEG into different frequency bands, and then took the maximum correlation between channel signals as the optimization objective to realize the feature extraction. The adaptive decomposition concept of the empirical mode decomposition (EMD) and the tight support framework of the wavelet transform (WT) are combined into empirical wavelet transform (EWT) (Gilles, 2013), which can recognize the location of the feature information in the Fourier spectrum and extract different frequency components of signals adaptively. Therefore, the EWT was used to decompose *n* channel signals in multi-channel EEG into different frequency bands, which would obtain *n* multi-dimensional signals composed of different bands components. The optimization objective was to maximize the covariance between *n* multi-dimensional signals, which aimed to extract the most relevant feature components from the frequency bands with common components in *n* channel signals. The Lagrange function was used to transform the optimization objective into a Rayleigh-Ritz eigenvalue problem whose solved values corresponded to the importance of extracted feature components. Besides, the feature extraction effects of PCA, ICA, and the proposed channel component correlation analysis (CCCA) methods were compared on multi-channel EEG. The results showed that the CCCA method could find the feature bands with common components between multi-channel EEG, and could better extract the effective feature components in multi-channel signals.

The organization of this paper is as follows. In Section II, the data and methods used in this paper are introduced. Section III analyzes the effect of CCCA method on the feature components extraction of multi-channel event-related potential (ERP) and steady-state visual evoked potential (SSVEP) signals, followed by discussions in Section IV. Finally, we conclude the work in Section V.

## 2 Methods and materials

### 2.1 Empirical wavelet transform

Through designing an adaptive wavelet filter, EWT can extracts a series of amplitude modulation-frequency modulation (AM-FM) single-component signals with compact support Fourier spectrum. The key to the design of adaptive wavelet filter is the frequency band division. EWT standardizes the frequency range of the Fourier spectrum to [0, *π*] based on Shannon criterion, and divides it into *N* segments using a frequency band division method. Each frequency band is expressed as *∇_n_* = [*w*_*n-*1_, *w_n_*] (*n* = 1, …, *N*), *w_n_* and *w*_*n-*1_ are the upper and lower limits of each frequency band, respectively. Based on the idea of constructing wavelets by Little wood-Paley and Meyer theory, the empirical scaling function *ϕ_n_*(*w*) as shown in Equation 1 and empirical wavelet function *γ_n_*(*w*) of wavelet transform shown in Equation 2 are defined respectively:


(1) $$\emptyset_{n}\left(w\right)=\{\begin{array}{c} 1\;\;\text{if}\;|w|\leq w_{n}-\tau_{n}\\ \cos\left\{\frac{\pi }{2}\beta \left[\frac{1}{2\tau_{n}}\left(|w|-w_{n}+\tau_{n}\right)\right]\right\}\;\\ \begin{array}{c} \;\text{if}\;w_{n}-\tau_{n}\leq |w|\leq w_{n}+\tau_{n}\\ 0\;\text{otherwise} \end{array} \end{array}$$

(2) $$\gamma_{n}\left(w\right)=\{\begin{array}{c} 1\;\text{if}\;w_{n}+\tau_{n}\leq |w|\leq w_{n+1}-\tau_{n+1}\\ \begin{array}{c} \cos\left\{\frac{\pi }{2}\beta \left[\frac{1}{2\tau_{n+1}}\left(|w|-w_{n+1}+\tau_{n+1}\right)\right]\right\}\;\\ \text{if}\;w_{n+1}-\tau_{n+1}\leq |w|\leq w_{n+1}+\tau_{n+1}\\ \begin{array}{c} \begin{array}{c} \sin\left\{\frac{\pi }{2}\beta \left[\frac{1}{2\tau_{n}}\left(|w|-w_{n}+\tau_{n}\right)\right]\right\}\\ \text{if}\;w_{n}-\tau_{n}\leq |w|\leq w_{n}+\tau_{n} \end{array}\\ 0\;\text{otherwise} \end{array} \end{array} \end{array}$$

where *w* is the frequency value, *w_n_* is the *n-*th boundary frequency, and *τ_n_ = μw_n_*, where *μ* is the transformation parameter to ensure that there is no overlap between *w_n-1_* and *w_n_*. *β(x)* is a polynomial that satisfies the Equation 3 and is defined in the interval [0, 1]:


(3) $$\beta \left(x\right)=x^{4}\left(35-85x+70x^{2}-20x^{3}\right)$$

Similar to WT, the approximate coefficient *W_s_^ε^(0,t)* and the detail coefficient *W_s_^ε^(n,t)* are obtained by the inner product of the signal with *ϕ_n_*(*w*) and *γ_n_*(*w*) respectively. The low and high frequency components of the signal are represented by the approximate coefficient and the detail coefficient. By using EWT, the signal *f(t)* will be decomposed into a series of AM-FM component *f_k_*(*t*) (*k* = 1, 2, …) with frequency from low to high as shown in Equations 4,5:


(4) $$f_{0}\left(t\right)=W_{s}^{\varepsilon }\left(0,t\right)*\emptyset_{1}\left(t\right)$$

(5) $$f_{k}\left(t\right)=W_{s}^{\varepsilon }\left(k,t\right)*\gamma_{k}\left(t\right)$$

where the symbol * denotes the convolution operation.

### 2.2 Channel component correlation analysis

**Figure 1:**
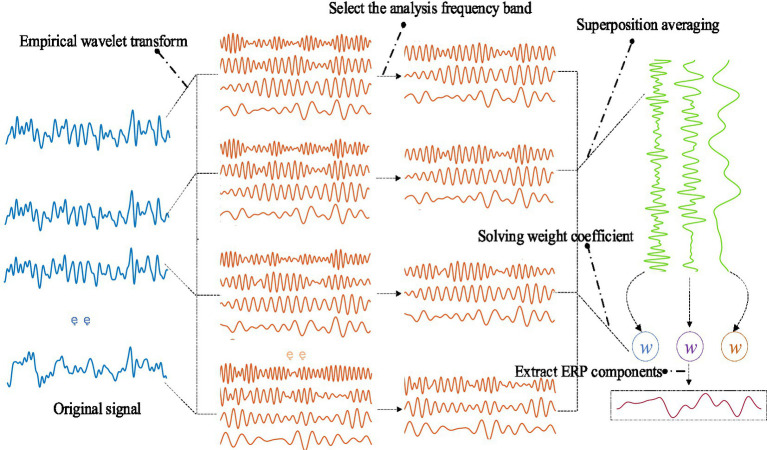
Channel component correlation analysis proced.

The procedure of CCCA proposed in this study is shown in Figure 1. To be specific, for the multi-channel EEG *X* = [*x_1_*, *x_2_*, *…*, *x_nc_*], where *n_c_* represents the number of channels, the EWT was used to decompose the each channel signal *x_i_* into different frequency bands. After decomposing *x_i_*, the frequency band decomposition results were reconstructed into *F_i_ = [f_i,1_, f_i,2_, …, f_i,m_](i* = *1, 2, …, nc)*, where *m* represented the number of frequency bands. In general, the effective feature components of signal are located in a specific frequency band. By giving a larger weight to the effective frequency band and a smaller weight to the non-effective frequency band, the main feature components can be extracted by linear summation as shown in Equation 6:


(6) $$s_{i}\left(t\right)=\sum_{j=1}^{m}w_{j}\cdot f_{i,j}$$

where *w_j_* is the weight coefficient corresponding to the *j*-th frequency band in the decomposed signal *F_i_*, and *s_i_(t)* is the effective feature component of the signal *x_i_*. The weight coefficient determines the extraction effect of the feature components of the EEG signal. In this paper, the maximum correlation between the effective feature components extracted from each channel was taken as the optimization goal, and the weight coefficient was solved to extract the common components in the multi-channel EEG signals. The sum of the covariances between the effective feature components extracted from each channel is shown in Equation 7:


(7) $$\sum_{k,l=1,k\neq l}^{n_{c}}cov\left(s_{k},s_{l}\right)=\sum_{k,l=1,k\neq l}^{n_{c}}\sum_{g,h=1}^{m}w_{g}w_{h}cov\left(f_{k,g},f_{l,h}\right)=W^{T}SW$$

where *S* is defined as shown in Equation 8:


(8) $$\sum_{k,l=1,k\neq l}^{n_{c}}\sum_{g,h=1}^{m}cov\left(f_{k,g},f_{l,h}\right)=\sum_{k,l=1,k\neq l}^{n_{c}}cov\left(F_{k},F_{l}\right)$$

When maximizing *W*^T^*SW* was used as the objective optimization function, the unique solution of *W* cannot be obtained. To obtain a finite solution, the effective feature components *s_i_*(*t*) extracted from each channel EEG signal need to be normalized as shown in Equation 9:


(9) $$\sum_{i=1}^{n_{c}}var\left(s_{i}\right)=\sum_{i=1}^{n_{c}}\sum_{g,h=1}^{m}w_{g}w_{h}cov\left(f_{i,g},f_{i,h}\right)=W^{T}QW=1$$

where *Q* is defined as shown in Equation 10:


(10) $$Q=\sum_{i=1}^{n_{c}}cov\left(F_{i},F_{i}\right)$$

Thus, the solution of *W* can be constructed as shown in Equation 11:


(11) $$W=\arg\max_{W}\frac{W^{T}SW}{W^{T}QW}$$

The equation above is a Rayleigh quotient problem of *S* and *Q*, and can be equivalent to Equation 12:


(12) $$\begin{array}{l} \min_{W}\;-W^{T}SW\\ s.t.\;W^{T}QW=1 \end{array}$$

Define a Lagrange function as shown in Equation 13 by introducing the Lagrange multiplier *λ*:


(13) $$L\left(w\right)=-W^{T}SW+\lambda \left(1-W^{T}QW\right)$$

Derivate *L(w)* to *W* and make it equal to 0, resulting in Equation 14:


(14) $$\frac{\partial L\left(W\right)}{\partial W}=SW-\lambda QW=0$$

Based on the equation *SW = λQW*, the closed-form solution of *W* is a matrix of eigenvectors obtained by the generalized eigenvalue decomposition of *Q^−1^S*. After obtaining the weight coefficients corresponding to each frequency band, the feature components can be extracted by the following formula Equation 15:


(15) $$y=W^{T}\cdot \frac{1}{n_{c}}\sum_{i=1}^{n_{c}}F_{i}$$

The pseudo code of the CCCA algorithm is shown as follows:


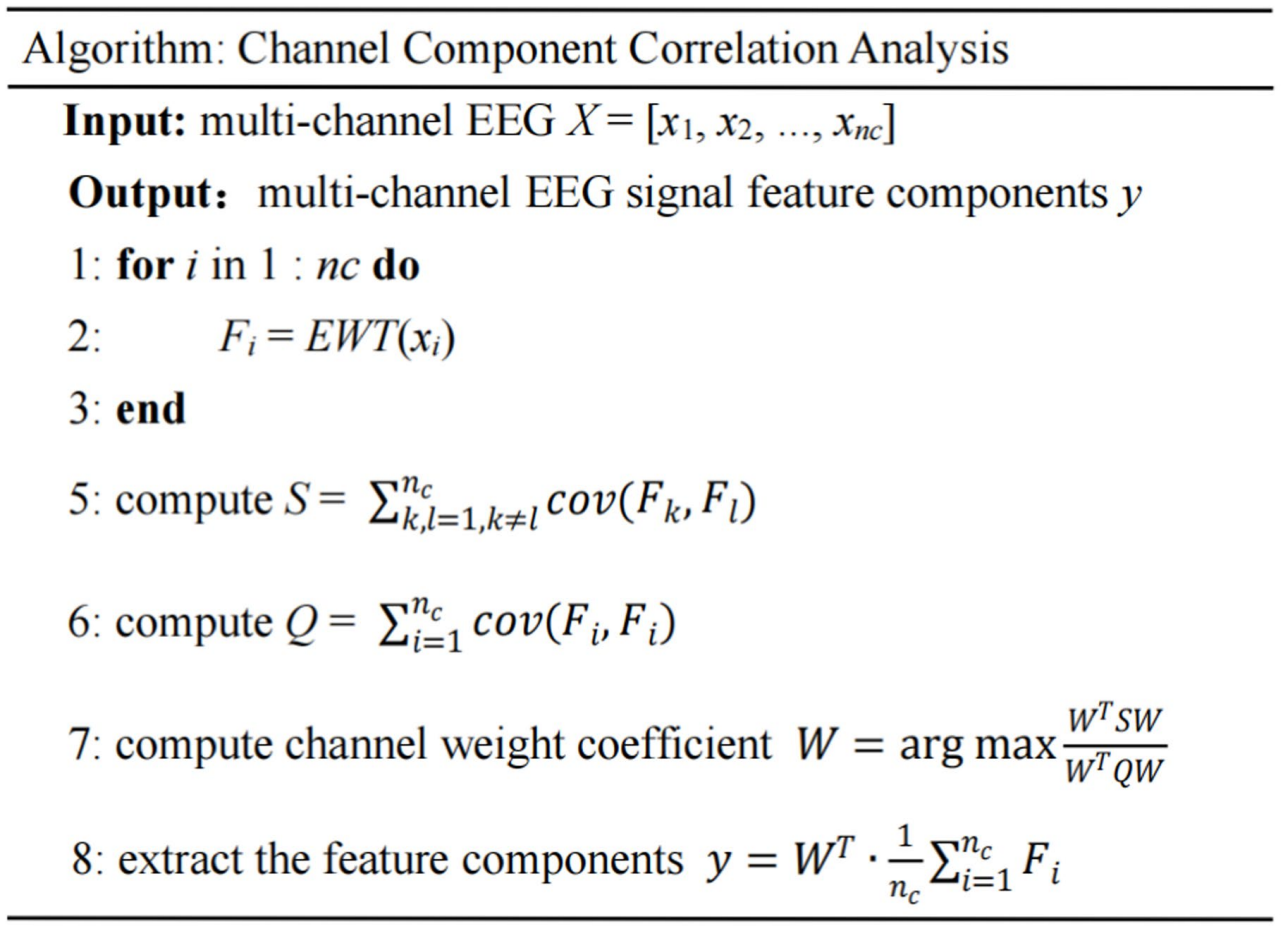


### 2.3 The data used in this study

The event-related potential (ERP) dataset (Zheng et al., 2020) used in this study is comprised of target image detection tasks, and the dataset is freely available at https://doi.org/10.6084/m9.figshare.12824771.v1. The stimulation was presented by a 24.5-inch liquid crystal display (LCD) monitor with a resolution of 1920 × 1,080 pixels and a vertical refresh rate of 60 Hz. Street scene images were presented at 10 Hz (10 images per second) in the center of the screen within a 1,200 × 800-pixel square. The images containing humans were regarded as target images and the subjects were asked to press keys immediately after they detected a target. The dataset includes 14 healthy subjects and the sample rate is 1,000 Hz. For each subject, the experiment consisted of three blocks. Each block contained 56 target image stimulus trials. The ERP signal analysis channels selected in this study were FP_2_, AF_3_, AF_4_, F_1_, F_Z_, F_2_, FC_1_, FC_Z_, FC_2_, C_1_, C_Z_, C_2_, P_1_, P_Z_, P_2_.

The steady-state visual evoked potential (SSVEP) dataset (Wang et al., 2017) used in this study is freely available on http://bci.med.tsinghua.edu.cn/download.html. This dataset includes SSVEP-BCI recordings of 35 healthy subjects focusing on 40 characters flickering at different frequencies (8–15.8 Hz with an interval of 0.2 Hz). For each subject, the experiment consisted of six blocks, where each block contained 40 trials corresponding to all 40 characters presented in a random order. The sampling frequency of the data is 250 Hz. The SSVEP signal analysis channels selected in this study were O_1_, O_2_, O_Z_, PO_3_, PO_4_, PO_Z_, PO_5_, and PO_6_.

## 3 Results

### 3.1 Analysis of the feature components extraction effect on ERP signals based on CCCA

#### 3.1.1 Layer selection of empirical wavelet transform decomposition

**Figure 2:**
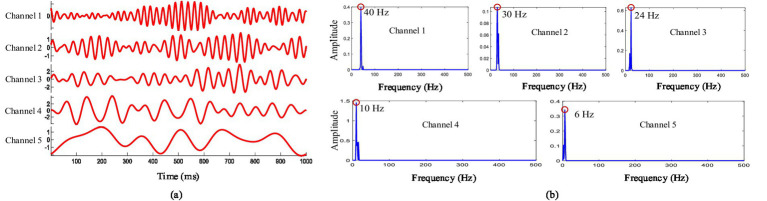
**(A)** Results of empirical wavelet decomposition. **(B)** Power spectrum analysis of empirical wavelet decomposition.

Empirical wavelet decomposition was applied by the CCCA method on each channel signal of the multi-channel EEG. Figure 2A shows the five-layer decomposition results of the channel FP_2_, in which each layer corresponds to a frequency level. Figure 2B shows the power spectrum corresponding to each layer, and it can be seen that the central frequency of the five-layer are mainly at 40 Hz, 30 Hz, 24 Hz, 10 Hz and 6 Hz, respectively. Considering that the feature components of ERP are at a low frequency, and only the decomposition results of the third to fifth layers were selected for subsequent feature components extraction.

#### 3.1.2 Analysis of the feature components extraction effect of single-trial ERP

**Figure 3:**
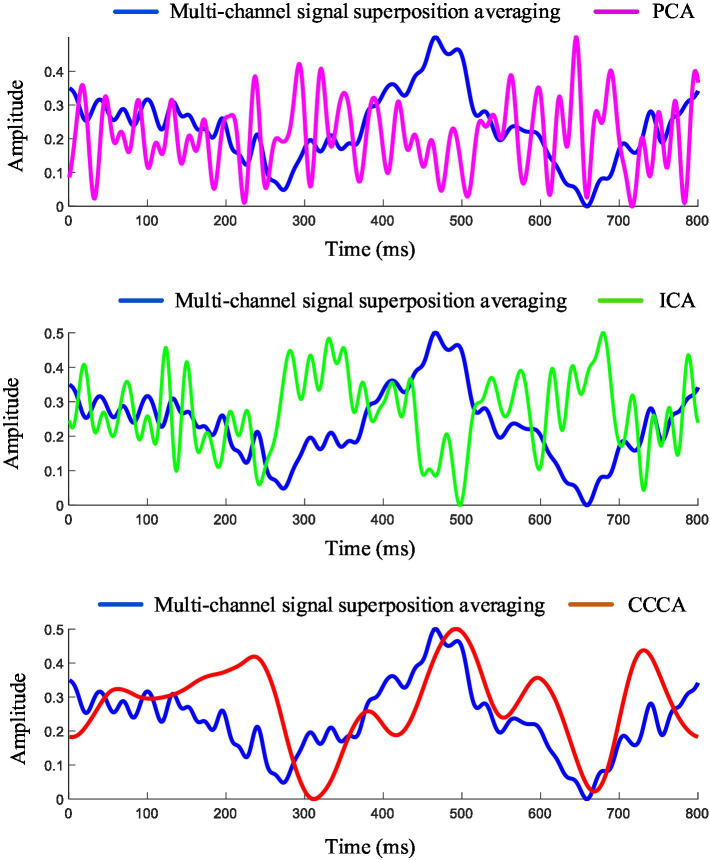
Feature components extraction results of single-trial ERP.

The ERP signals were extracted with 800 ms after the target image appeared, filtered by a [2, 50] Hz band-pass filter. Figure 3 shows the ERP feature components extracted from channels (FP_2_, AF_3_, AF_4_, F_1_, F_Z_, F_2_, FC_1_, FC_Z_, FC_2_, C_1_, C_Z_, C_2_, P_1_, P_Z_, P_2_) using PCA, ICA and CCCA methods. To compare the extraction effects of the three methods, the ERP signals obtained by 56 target image stimuli in electrode F_Z_ were superposed and averaged as the reference signal. It can be seen from Figure 3 that the signal-to-noise ratio (SNR) of ERP is effectively improved after superimposing and averaging, and the P300 feature component can be significantly observed. In the extraction results of ERP induced by single-trial target images stimuli, PCA hardly extracted effective ERP components, and the result of ICA was quite different from the reference signal, while the result of CCCA was highly similar to the reference signal in terms of latency and amplitude. According to the results of PCA and ICA, it is difficult to extract effective feature components of single-trial evoked EEG due to their weak amplitude and large background noise. Although the SNR of multi-trial ERP signals can be improved by the superposing and averaging method, it also causes the loss of dynamic variation information of ERP signals across trials (Mouraux and Iannetti, 2008). Therefore, the precise feature component extraction method of single-trial EEG has attracted much attention. Compared with the commonly used PCA and ICA methods, the CCCA method proposed in this study has important engineering application value in the feature components extraction of single-trial ERP.

**Figure 4:**
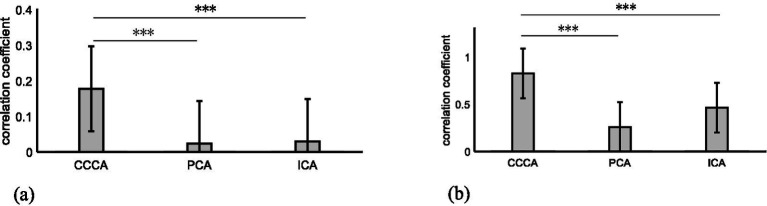
**(A)** The similarity results of the ERP feature waveforms extracted, respectively, by the three methods of CCCA, PCA, and ICA and the reference signal in single-trial multi-channel EEG signals. **(B)** The similarity results of the ERP feature waveforms extracted, respectively, by the three methods of CCCA, PCA, and ICA and the reference signal in multi-trial single-channel EEG signals.

Based on the similarity between the ERP feature waveforms extracted by the CCCA, PCA, and ICA methods and those derived from the reference signal (The ERP signals obtained from channel Fz under 56 stimulations of the target image were superimposed and averaged to serve as the reference signal) as the judgment criterion, this paper compared the extraction effects of these three methods on the ERP feature components of single-trial multi-channel EEG signals. We calculated the similarity results for all trials of each subject and then carried out a superimposed average on the calculation results of all subjects. As shown in Figure 4A, it can be observed that the ERP feature waveforms extracted by the CCCA method exhibit the strongest correlation with those obtained from the reference signal. Moreover, the ANOVA test indicates that there are significant differences between the extraction effect of the CCCA method on ERP feature components and that of PCA as well as ICA (***p < 0.001).

#### 3.1.3 Analysis of the feature components extraction effect of multi-trial ERP of the same electrode

**Figure 5:**
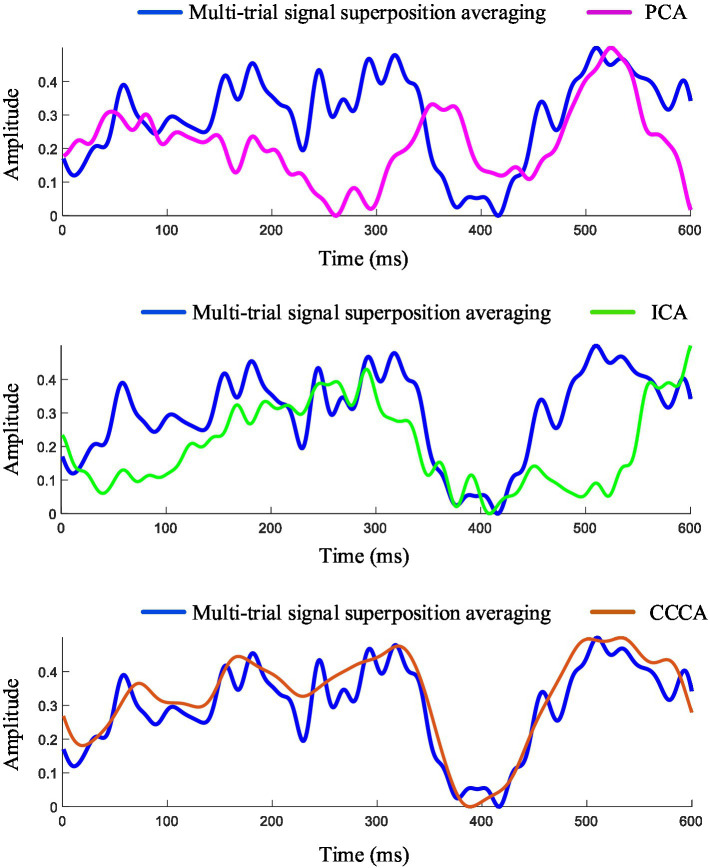
Feature components extraction results of multi-trial ERP of the same electrode.

The multi-trial average method is to superpose and average the multi-trial ERP signals of the same electrode. The multi-trial ERP can be constructed into a multi-channel form by regarding each trial signal as a channel signal. Figure 5 shows the ERP feature components extracted by applying PCA, ICA, CCCA and multi-trial average methods on EEG signals, which were obtained by 25 target images stimuli in FC_Z_ electrode. It can be seen from Figure 5 that the extraction effect of PCA was not ideal, which was quite different from the results of the multi-trial average method, the results of ICA had a certain similarity with that of the multi-trial average method, and CCCA achieved the most similar extraction results. In addition, compared with the multi-trial average method, the ERP waveform extracted by CCCA method was smoother, which would be more conducive to confirming parameters such as the latency and amplitude of the P300 component. Likewise, we adopted the similarity between the ERP feature waveforms extracted via the CCCA, PCA, and ICA methods and those derived from the reference signal (The EEG signals obtained from electrode channel FCz under 25 stimulations of the target image.) as the judgment criterion. Subsequently, we computed the similarity results for each subject and conducted a superimposed average on the calculation results of all subjects. As depicted in Figure 4B, it is evident that the ERP feature waveforms extracted by the CCCA method from the signals of the same electrode channel across multiple trials possess the highest correlation with the ERP feature waveforms obtained from the reference signal. Moreover, the ANOVA test demonstrates that there are significant differences between the extraction effect of the CCCA method on ERP feature components and those of PCA and ICA (***p < 0.001).

Comparing the results of Figures 3, 5, it can be seen that the ERP components extraction effect of the multi-trial signals of the same electrode was better than that of the single-trial signals composed of different electrodes. This may because different electrodes do not all have good EEG responses, and channel selection is needed to achieve better results. It may also because different electrodes have different phases in a single trial, but the phase of the same electrode is locked in different trials, so the effective feature components of EEG can be extracted more stably. Good performance has been shown by the CCCA method in single-trial and multi-trial ERP feature components extraction, indicating that it has potential application value in multi-channel EEG feature components extraction.

#### 3.1.4 Analysis of common feature components extraction effect of multi-channel EEG based on the CCCA method

**Figure 6:**
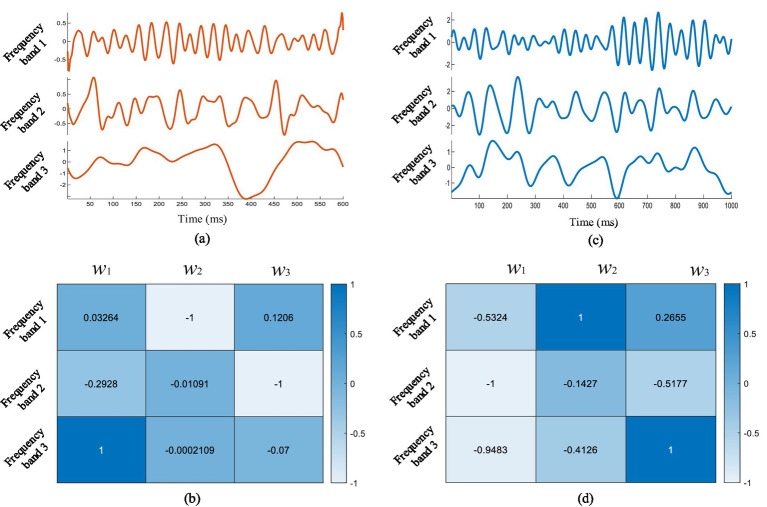
**(A)** The superposition average results of empirical wavelet decomposition of multi-channel EEG under target image stimulation, and **(B)** the weight coefficients of each frequency band solved by CCCA method. **(C)** The superposition average results of empirical wavelet decomposition of multi-channel EEG under non-target image stimulation, and **(D)** the weight coefficients of each frequency band solved by CCCA method.

As shown in Figure 1, the CCCA method first applied EWT on each channel signal and decomposed it into different frequency bands. Then, the maximum correlation between the effective feature components extracted from each channel EEG signal was taken as the optimization goal, in order to solve the corresponding weight coefficients of each frequency band. Figure 6A showed the superposition average results of the 3–5 layer empirical wavelet decomposition (corresponding to Equation 15) obtained from ERP signals of the channels (FP_2_, AF_3_, AF_4_, F_1_, F_Z_, F_2_, FC_1_, FC_Z_, FC_2_, C_1_, C_Z_, C_2_, P_1_, P_Z_, P_2_), which were induced by target images stimuli. It can be seen from Figure 6A that the frequency band 3 had a strong similarity with the ERP waveform, but frequency bands 1 and 2 did not have obvious ERP components. The effective ERP feature components can be extracted by giving a larger weight to frequency band 3 and a smaller weight to frequency bands 1 and 2. Figure 6B showed three sets of weight coefficients calculated by the CCCA method, which corresponded to the feature importance from large to small, and we only used the first set to extract EEG feature components. It can be seen from Figure 6B that the weight coefficient *w_1_* would give the maximum weight to frequency band 3, and the frequency band 1 and 2 would be given a smaller weight. This indicated that the CCCA method could give a larger weight to the frequency bands with strong correlation of each channel, while the feature bands with weak correlation of each channel were given a smaller weight, so as to extract the common feature components in the multi-channel EEG.

We also analyzed the common feature components extraction effect of multi-channel EEG under non-target image stimulation based on the CCCA method, and the results were shown in Figures 6C,D. It can be seen from Figure 2B that the center frequency of frequency band 1, band 2 and band 3 were 24 Hz, 10 Hz and 6 Hz, respectively. To extract the 10 Hz components induced by non-target images stimulation, frequency band 2 needed to be given a larger weight. Figure 6D was the weight coefficients of each frequency band obtained by the CCCA method, and the frequency band 2 was given the maximum weight. The above results fully proved the effectiveness of the CCCA method for the common feature components extraction of multi-channel signals.

### 3.2 Analysis of feature components extraction effect of SSVEP signals based on the CCCA method

**Figure 7:**
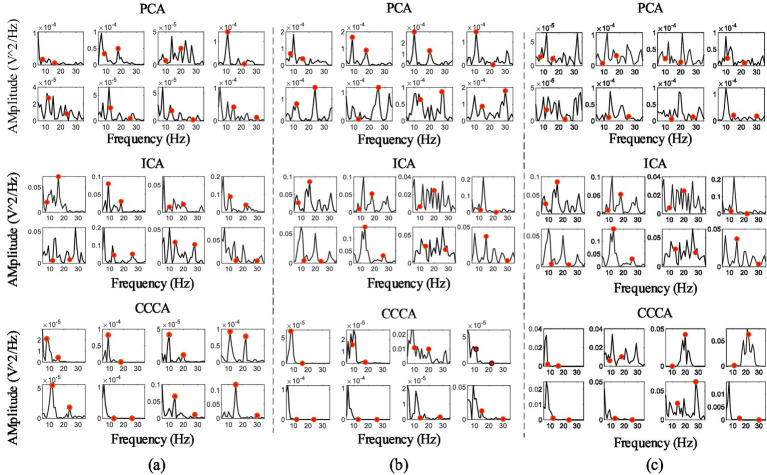
**(A–C)** The power spectrum of SSVEP feature components extracted by PCA, ICA and CCCA on subject 3, subject 4 and subject 6, respectively.

We also verified the CCCA method on the feature components extraction effect of multi-channel SSVEP, whose frequencies were 8 Hz, 9 Hz, 10 Hz, 11 Hz, 12 Hz, 13 Hz, 14 Hz and 15 Hz with 1 s data length, filtered with a [2, 50] Hz band-pass filter. Figures 7A–C were the power spectrum of SSVEP feature components in the channels (O_1_, O_2_, O_Z_, PO_3_, PO_4_, PO_Z_, PO_5_, and PO_6_) extracted by PCA, ICA, and CCCA using the data of subject 3, 4, and 6. The red dots in the figures marked the amplitude of the real frequency. As shown in Figure 7, the CCCA, PCA and ICA methods achieved the best feature components extraction effect in subject 10, subject 6 and subject 4, respectively, which indicated that different subjects had individual differences, and one method cannot be applied to all subjects. Differences in physiological structures are likely to be one of the contributing factors to the disparities in individual electroencephalogram (EEG) signals. Specifically, variations exist among individuals with regard to the folding extent of the cerebral cortex, the depth of sulci and gyri, as well as the relative dimensions and configurations of diverse brain regions. These distinct characteristics exert a significant influence on the distribution and the patterns of interconnection among neurons. Consequently, discrepancies among individuals manifest themselves in the processes of EEG signal generation and conduction. When it comes to EEG signals themselves, individual differences can be observed in aspects such as the amplitude and frequency components of the signals.

We took the frequency corresponding to the highest amplitude value of the power spectrum as the criterion for determining whether it was the real stimulation frequency, and based on this, calculated the recognition accuracy rates of all subjects (except subject 5 whose data was damaged) in the dataset under the PCA, ICA, and CCCA methods. The results are presented in Table 1. As can be seen from the table, 12 subjects (marked with *) achieved the best recognition accuracy rates under the CCCA method. Specifically, for subject 26, the recognition accuracy rate of the CCCA method was higher than that of the PCA and ICA methods by 45.84 and 31.25%, respectively. For the remaining subjects in the dataset, the best recognition accuracy rates were obtained under the PCA or ICA method. Evidently, when the PCA and ICA methods fail to achieve ideal feature extraction effects on certain data, the CCCA method might yield better results. Therefore, for the feature components extraction of multi-channel SSVEP, CCCA method can form a good complement with PCA and ICA methods, becoming an important supplement when PCA and ICA fail.

**Table 1:** The recognition accuracy rates of SSVEP features under the CCCA, PCA, and ICA methods.

Subject	Accuracy			Subject	Accuracy		
	PCA	ICA	CCCA		PCA	ICA	CCCA
S1	0.4375	0.2708	0.1875	S19	0.1667	0.2083	0.1250
S2	0.2708	0.1458	0.1458	S20	0.3542	0.1875	0.2500
S3	0.3750	0.3333	0.3333	S21	0.2083	0.1667	0.1667
S4	0.3542	0.3750	0.1458	S22	0.3333	0.4375	0.5833*
S6	0.3750	0.3125	0.2500	S23	0.1667	0.1667	0.1042
S7	0.1458	0.2500	0.2708*	S24	0.3125	0.2500	0.3958*
S8	0.2708	0.2292	0.2292	S25	0.2083	0.5417	0.5625*
S9	0.1250	0.1250	0.1667*	S26	0.2708	0.4167	0.7292*
S10	0.2708	0.3333	0.4167*	S27	0.2500	0.2500	0.1458
S11	0.0833	0.1875	0.1250	S28	0.3333	0.1250	0.1875
S12	0.2292	0.3333	0.3333*	S29	0.1042	0.2917	0.1458
S13	0.2917	0.2917	0.1667	S30	0.1875	0.1042	0.2083*
S14	0.6250	0.7083	0.6875	S31	0.6250	0.5208	0.4375
S15	0.2500	0.4167	0.6250*	S32	0.4167	0.3333	0.3958
S16	0.2083	0.2292	0.1667	S33	0.1042	0.1458	0.1250
S17	0.5000	0.3750	0.2292	S34	0.5625	0.4167	0.6250*
S18	0.3750	0.3125	0.2083	S35	0.2292	0.3125	0.4167*

## 4 Discussion

EEG corresponds to electrophysiological activity on the cerebral cortex or scalp’s surface and is widely used clinically to detect brain electrical activity. The accurate analysis of EEG signals is required for the effectiveness of this important diagnostic tool.

EEG signals are generally recorded as multi-channel time-varying data, as single-channel EEG cannot obtain information from multiple brain regions. Electrodes that respond best for different applications may vary due to differences in the surroundings or the shifting of electrodes, so it is difficult to obtain robust results using only a single electrode channel. Therefore, the analysis of multi-channel EEG signals is crucial for effective EEG research. With inevitable information redundancy in multi-channel EEG signals, signal-processing methods must be used to identify active components and remove noise and redundancy. Both PCA and ICA methods are commonly used for multi-channel signal feature component extraction. PCA assumes that the source signals and noise are not statistically correlated, and ICA assumes for a statistical independence relationship of source signal and noise, so ICA generally performs considerably better than PCA. However, an additional step is required in ICA to select the useful components. Aiming to overcome the limitations of the existing analysis methods, we developed a new method for multi-channel EEG feature component extraction.

The effective feature components are normally located in a specific frequency band, so we can extract them by giving larger weight to the feature band and smaller weight to the noise band after decomposing the signal into different frequency bands. Considering that the EWT can recognize the location of the feature information in the Fourier spectrum with a compactly supported wavelet filter, and adaptively extract different frequency components, each electrode channel signal in the multi-channel EEG is decomposed into different frequency bands through the EWT. Multi-channel EEG signals are collected at the same time, so each electrode channel contains signal components. Therefore, the signal components have a strong correlation between channels, while the noise components have a weak correlation. Based on this, the maximization of the covariance between the feature components extracted from each channel is taken as the optimization objective, and the weight coefficients corresponding to each frequency band are solved.

The traditional cross-trial average method is widely used to enhance the SNR of EEG in order to extract ERP components. However, the loss of lock-time non-lock-phase signals (such as event-related synchronization or event-related desynchronization) will be caused by this method, and also the loss of cross-trial variation information of ERP (Mouraux and Iannetti, 2008). How to improve the SNR of single-trial ERP is a research project with remarkable attention. In this study, the superposition average results of multi-trial ERP was used as benchmarks, and the feature components extraction effects of PCA, ICA and CCCA method were compared on single-trial multi-channel ERP, which indicates that CCCA method could achieve the most similar extraction effect as the benchmarks. We also analyzed the feature components extraction effect of the CCCA method on the multi-trial ERP of the same electrode and the EEG signals induced by non-target image stimulation, which indicated that the CCCA method is superior to PCA and ICA methods. Besides, the extraction effects of PCA, ICA and CCCA methods were compared for multi-channel SSVEP signals. It was found that due to the existence of individual differences, one method was not suitable for all subjects. When PCA and ICA methods failed in some data analysis, the CCCA method may be effective and can be used as an important method supplement. We verified that the CCCA method could find the feature frequency band with strong correlation between EEG channels, so as to give it a large weight. By assigning appropriate weight coefficients to the frequency bands obtained by EWT, the common components in multi-channel EEG can be effectively extracted, which shows a good application prospect in multi-channel signal feature extraction.

The methodology put forward in this paper has exhibited favorable performance in extracting the ERP feature components from multi-channel signals within a single trial and also in extracting the ERP feature components from the signals of the same electrode channel across multiple trials. In forthcoming research endeavors, we intend to apply this methodology to the online recognition within the brain-computer interface system based on rapid serial visual presentation (RSVP) (Zheng et al., 2020). Through precisely extracting the EEG ERP features triggered by target image stimuli, we strive to accomplish the rapid and accurate recognition of target images.

## 5 Conclusion

To effectively remove the noise and information redundancy and extract the effective feature components in multi-channel EEG signals, a channel component correlation analysis method is proposed in the paper. First, each electrode signal of the multi-channel EEG signal is decomposed into different frequency bands by the empirical wavelet transform. Then, through taking the maximization of the covariance of the feature components extracted from each channel as the optimization goal, the feature frequency bands with strong correlation are given a larger weight, while the feature frequency bands with weak correlation are given a smaller weight, so as to extract the common components in the multi-channel EEG. Compared with PCA and ICA methods, the excellent performance of CCCA method in multi-dimensional signal feature components extraction shows great application and research value.

## Data Availability

The datasets presented in this study can be found in online repositories. The names of the repository/repositories and accession number(s) can be found below: https://doi.org/10.6084/m9.figshare.12824771.v1.
